# A comparison of an objective and subjective test of stress urinary incontinence (SUI) and their acceptability to participants

**DOI:** 10.1186/1745-6215-16-S2-P58

**Published:** 2015-11-16

**Authors:** Tracey Davidson, Alison McDonald, Gladys McPherson, John Norrie

**Affiliations:** 1 University of Aberdeen, Aberdeen, UK

## Background

SUI is a common and distressing condition affecting 3 million women in the UK. As part of the multicentre NIHR HTA funded SIMS study (Adjustable Anchored Single-Incision Mini-Slings Versus Standard Tension-Free Mid-Urethral Slings in the Surgical Management Of Female Stress Urinary Incontinence; A Pragmatic Multicentre Non-Inferiority Randomised Controlled Trial) we are evaluating the acceptability and correlation of two patient tests of SUI -the objective 24hr pad test (PT) and the subjective home continence stress test (HCST).

## Method

Participants (n=197) were given sufficient standardised, pre-weighted incontinence pads to wear for 24hrs (changing when necessary) and two large tissue sheets. Used pads were returned to the study team in sealed plastic bags on the day of surgery for weighing and disposal. The tissue sheets were used as part of the HCST conducted before and after the pad test. Participant feedback on both tests is being collected.

## Results

Results will be presented reporting response rates to both tests and comparisons between responses to the PT and the HSCT.

## Discussion

The acceptability to participants and the advantages and disadvantages of each test from a trial management perspective will be discussed. Data collection challenges will be considered including; issuing of pads to sites and participants, the receipt (including the handling and delivery) of used pads and the decision process around which data to use in analysis when responses are available to both tests. Cost implications of both tests will also be presented.

